# RNA Interference Targeting *MaCht-2* Induces Severe Molting Defects and Lethality in *Monochamus alternatus*

**DOI:** 10.3390/insects17050530

**Published:** 2026-05-21

**Authors:** Siming Fang, Xiaoxiao Chang, Han Chen, Juan Shi

**Affiliations:** 1 Beijing Key Laboratory Forest Pest Control, Beijing Forestry University, Beijing 100083, China; ming1607@126.com (S.F.); changxx2021@163.com (X.C.); chenhan20020410@163.com (H.C.); 2 Sino-French Joint Laboratory for Invasive Forest Pests in Eurasia, Coll Forestry, Being Forestry University, Beijing 100083, China

**Keywords:** *Monochamus alternatus*, RNA interference, *Macht-2*, molting, pest management

## Abstract

*Monochamus alternatus*, the principal vector of pine wilt disease, poses a serious threat to pine forest ecosystems, and the identification of effective molecular targets is important for the development of environmentally friendly control strategies. In this study, a Group VII chitinase gene, *MaCht-2*, was identified in *M. alternatus*, and its developmental expression pattern, RNA interference (RNAi) efficiency, and RNAi-induced phenotypes were investigated using quantitative PCR, dsRNA injection, and transmission electron microscopy. *MaCht-2* was highly expressed from the fifth-instar larval stage to the pupal stage, indicating a role in molting and metamorphosis. Silencing of *MaCht-2* caused severe developmental defects across multiple life stages, including incomplete ecdysis, pupation failure, adult deformities, impaired locomotion, and high mortality. In third-instar larvae, injection of 5 μg of dsRNA resulted in mortality exceeding 50%. Ultrastructural analysis further showed that *MaCht-2* silencing disrupted normal cuticle organization, causing thinning, wrinkling, and disordered lamellar structure. Compared with previously studied chitin-related genes, *MaCht-2* produced stronger lethality and more stable RNAi effects. These results indicate that *MaCht-2* is essential for normal development of *M. alternatus* and represents a promising target for RNAi-based pest management.

## Simple Summary

*Monochamus alternatus* is the main vector of pine wilt disease and plays a critical role in the spread of this destructive forest disease. Developing efficient and environmentally friendly strategies to control this insect is therefore of great importance. Chitinases are key enzymes involved in insect molting and cuticle formation and are considered promising targets for RNA interference (RNAi)-based pest control. In this study, we characterized *MaCht-2*, a Group VII chitinase gene in *M. alternatus*, and evaluated its function during development. We found that *MaCht-2* was highly expressed during the fifth-instar larval and pupal stages. Silencing *MaCht-2* caused severe molting failure, developmental abnormalities, impaired locomotion, and high mortality in larvae, pupae, and adults. In addition, cuticle ultrastructure was seriously disrupted after gene knockdown. These results show that *MaCht-2* is essential for normal development of *M. alternatus* and suggest that it is a promising molecular target for RNAi-based management of this important forest pest.

## 1. Introduction

*Monochamus alternatus* is a major vector of pine wilt disease (PWD), a destructive forest pathology that threatens pine ecosystems globally due to its rapid transmission and high mortality rates [1,2,3]. Adult beetles effectively spread pine wood nematodes during feeding and oviposition, while larval feeding within host trunks further compromises tree vigor and structural integrity [4,5]. Current *M. alternatus* control strategies primarily depend on chemical insecticides and physical control measures, which are often associated with ecological risks and limited target specificity [6,7]. Consequently, the development of environmentally sustainable and target-specific molecular control approaches has become increasingly imperative. Because *M. alternatus* functions as a vector rather than merely a defoliating or wood-boring pest, targets that impair emergence quality, locomotion, and reproductive performance may be epidemiologically valuable even when mortality is incomplete.

Insect molting and metamorphosis are critical developmental processes governed by cuticle cyclical degradation and reconstruction. Chitin, a principal structural component of the insect exoskeleton, undergoes continuous remodeling mediated by various enzymes involved in chitin metabolism [8,9]. Chitinases are essential for the degradation of old cuticles and formation of new cuticles [8,10,11]. Disruption of genes associated with chitin metabolism often results in molting abnormalities, defective cuticle formation, and insect mortality, underscoring their significance as potential molecular targets for pest management [8,12]. However, chitin metabolism is regulated by a complex enzymatic network, and different chitin-related genes may contribute unequally to cuticle turnover, structural maintenance, and developmental transitions. As a result, silencing different components of this pathway can lead to markedly different biological outcomes, ranging from mild morphological abnormalities to severe lethality. Therefore, the evaluation of chitin-related genes for pest management should not be limited to pathway-level annotation, but should also consider the relative functional importance and RNAi responsiveness of individual genes [8,13].

RNA interference (RNAi) has emerged as a potent strategy for functional genomics and pest management owing to its high target specificity and environmental safety [14,15,16]. Previous studies have demonstrated the applicability of RNAi in *M. alternatus*, where silencing of various functional genes resulted in developmental defects and mortality. Nevertheless, increasing evidence indicates that RNAi performance differs substantially among genes, even within the same biological pathway, and that transcript knockdown does not always translate into equally strong or persistent phenotypic consequences. Consequently, the central challenge is not merely whether RNAi can function in a given insect species, but which endogenous genes can serve as the most effective and reliable targets for control [13]. This issue is particularly important for chitin metabolism-related genes, because functional redundancy, developmental stage specificity, and differences in protein turnover may all influence the final biological outcome after RNAi treatment [8,13]. However, increasing evidence indicates that RNAi performance differs substantially among genes, even within the same metabolic pathway, resulting in lethality and phenotypic stability variability [13,17]. Therefore, identifying highly responsive RNAi targets is a crucial step in advancing practical molecular control strategies for this pest species. Thus, the key challenge is no longer whether RNAi can function in *M. alternatus*, but which endogenous targets yield the most robust, persistent, and biologically consequential responses.

Although chitinases are divided into multiple phylogenetic groups, the functional roles of Group VII chitinases remain unclear. Earlier studies have characterized two chitin-associated genes, *MaCht-3* and *MaIDGF-4*, in *M. alternatus*, and their silencing induced molting defects and developmental abnormalities [18]. Nonetheless, the observed differences in lethality and RNAi persistence among these genes indicated that individual components of the chitin metabolic network may contribute unequally to regulatory processes [19], highlighting the need to identify more effective target genes. In this context, the study of additional chitinase family members is important not only for clarifying their developmental functions, but also for prioritizing candidate RNAi targets with stronger and more stable biological effects [13]. Group VII chitinases are of particular interest because available evidence suggests that they are involved in insect development and eclosion, yet their functional characterization remains limited compared with that of several other chitinase groups. For example, knockdown of the Group VII chitinase gene *MdCht2* in *Musca domestica* caused high mortality and abnormal eclosion, supporting the view that *Cht2* genes can play important roles in cuticle remodeling and metamorphosis [20].

In this study, a Group VII chitinase gene, *MaCht-2*, was examined to determine its suitability as an efficient RNAi target. Its developmental expression dynamics, RNAi effectiveness, dose-dependent phenotypes, and alterations in cuticle ultrastructure were systematically evaluated. Rather than simply validating another chitin-related gene, this study sought to determine whether *MaCht-2* represents a functionally important and practically superior target within the chitin metabolic pathway of *M. alternatus* [13]. This study aimed to establish whether *MaCht-2* serves as a critical regulator of molting and cuticle formation and to evaluate its potential utility as a candidate target for *M. alternatus* RNAi-based control.

## 2. Materials and Methods

### 2.1. Insect Collection and Rearing

*M. alternatus* larvae were obtained from pine forests in Quannan County, Jiangxi Province, China. Field-collected larvae were transported to the laboratory immediately after sampling and maintained under controlled conditions to minimize stress before rearing and experimentation. Specimens were promptly transported to the laboratory and maintained in complete darkness under controlled environmental conditions at 25 °C with 60–70% relative humidity. Larvae were reared on an artificial diet provided by the Chinese Academy of Forestry until the desired developmental stages were reached for subsequent experiments. Individuals used for gene expression analysis and RNAi assays were continuously reared under the same environmental conditions until they reached the required developmental stages, including third-instar larvae, fifth-instar larvae, pupae, and newly emerged adults. Larval instars were determined based on the developmental criteria described by Huang et al. [21]. Only healthy and normally developing individuals were selected for subsequent experiments.

### 2.2. Screening and Identification of Target Genes

Target gene selection was performed using a previously established *M. alternatus* transcriptomic dataset. Keyword searches using “chitinase” within the annotated transcriptome database were used to retrieve candidate chitinase-related sequences. Next, the identified sequences were analyzed using BLASTX (version 2.16.0, BLAST+ 2.16.0, https://blast.ncbi.nlm.nih.gov/doc/blast-news/2024-BLAST-News.html, accessed on 24 May 2025) against the National Center for Biotechnology Information (NCBI) non-redundant protein database to confirm gene identity and evaluate sequence homology with known insect chitinases. Sequences with clear annotation support and high similarity to reported chitinase genes were selected as candidate targets for further characterization. The sequence ultimately analyzed in this study was identified as a Group VII chitinase gene and designated *MaCht-2*.

### 2.3. Protein Sequence and Structural Characterization

The open reading frames (ORFs) of the target gene were predicted using the ORF Finder tool (NCBI, https://www.ncbi.nlm.nih.gov/orffinder/, accessed on 12 November 2024) provided by NCBI. To examine protein domain organization, conserved domains were identified using the simple modular architecture research tool (SMART) (version 10.0, https://smart.embl.de, accessed on 12 November 2024) program on the European Molecular Biology Laboratory (EMBL) platform. Physicochemical parameters, including theoretical molecular weight and isoelectric point, were computed using the ProtParam tool (version 1.0, https://web.expasy.org/protparam/, accessed on 12 November 2024) of the ExPASy server. The deduced amino acid sequence was further examined to determine whether *MaCht-2* contained conserved catalytic and chitin-binding domains characteristic of insect chitinases. These analyses were used to provide a molecular basis for subsequent functional interpretation of *MaCht-2* in development and molting.

### 2.4. Expression Analysis at Different Developmental Stages

To characterize the developmental expression pattern of the target gene, epidermal tissues were sampled from first- to fifth-instar larvae (L1–L5), early pupae (2 days, EP), late pupae (8 days, LP), early adults (2 days, EA), and late adults (8 days, LA). Total RNA was isolated using a commercial RNA extraction kit (Yeasen Biotechnology, Shanghai, China). First-strand Complementary deoxyribonucleic acid (cDNA) was subsequently synthesized using a reverse transcription kit (Takara Bio Inc., Kusatsu, Shiga, Japan). Three biological replicates were prepared for each developmental stage.

Gene-specific primers for the target gene and the reference gene (β-actin) were designed using Primer-BLAST (Table 1) and synthesized by Tsingke Biotechnology (Beijing, China). Quantitative real-time PCR (qPCR) was performed using a CFX Connect real-time PCR System (Bio-Rad Laboratories, Hercules, CA, USA). The reaction mixture (20 µL) consisted of 10 µL of 2× Talent qPCR Premix, 0.8 µL of each primer (10 µmol/L), 2 µL of cDNA template, and nuclease-free water. Each reaction was performed under identical amplification conditions, and a melting curve was generated to verify amplification specificity.

The amplification protocol included an initial denaturation at 95 °C for 30 s, followed by 40 cycles of 95 °C for 5 s, 55 °C for 30 s, and 72 °C for 30 s. A melting curve analysis was performed at the end of the amplification process. Relative gene expression levels were determined using the 2^−ΔΔCt^ method. Statistical analyses were performed using the Prism version 8.3.0 software. The expression level at one selected developmental stage was used as the calibrator for relative comparison among stages.

### 2.5. Double-Stranded RNA Synthesis and RNAi Treatment

Gene-specific fragments were amplified using primers incorporating T7 promoter sequences (TAATACGACTCACTATAGGG) (Table 1). The resulting PCR products served as templates for the in vitro synthesis of double-stranded RNA (dsRNA) using a T7 RNAi Transcription Kit (Vazyme, Nanjing, China).

The synthesized dsRNA was purified by precipitation with 0.1 volume of 3 M sodium acetate (pH 5.2) and 1 volume of isopropanol, followed by centrifugation and washing with 70% cold ethanol. The purified dsRNA was resuspended in nuclease-free water and quantified. The dsRNA targeting *MaCht-2* was designated ds*MaCht-2*, whereas dsRNA targeting green fluorescent protein (GFP) was used as the control.

Three dosage levels (5 µg, 10 µg, and 15 µg dsRNA) were administered to third- and fifth-instar larvae, and pupae using a Hamilton microsyringe (Hamilton Central Europe SRL, Timisoara, Romania). A minimum of five individuals were injected per treatment group for phenotypic assessment. Individuals injected with an equivalent dose of double-stranded green fluorescent protein (dsGFP) were used as controls. Following injection, insects were returned to the same rearing conditions described above and monitored continuously for survival, molting success, and morphological abnormalities. Phenotypic changes were recorded throughout subsequent development according to the stage at which dsRNA was administered.

### 2.6. Quantification of Gene Expression After RNAi Treatment

To evaluate the silencing efficiency of ds*MaCht-2*, epidermal tissues were sampled at 12 h, 24 h, and 48 h after dsRNA injection at different concentrations (5 µg, 10 µg, and 15 µg). Total RNA extraction, cDNA synthesis, and qPCR procedures were performed as described in Section 2.4. Each treatment included three biological replicates, and insects injected with dsGFP served as controls. Relative expression levels were calculated by comparing ds*MaCht-2*-treated insects with the corresponding dsGFP-treated group at each time point, thereby allowing assessment of both the intensity and duration of RNAi-mediated suppression.

### 2.7. Transmission Electron Microscopy (TEM)

To examine ultrastructural changes in the cuticle after *MaCht-2* silencing, elytral and abdominal epidermal tissues were collected from newly emerged adults derived from insects injected with 5 µg ds*MaCht-2* or dsGFP. These two tissue types were selected to compare the effects of gene silencing on cuticle organization in different adult body regions.

Tissue samples (~1 mm^3^) were fixed overnight in 2.5% glutaraldehyde at 4 °C and then post-fixed for 1–2 h in a solution containing 1% osmium tetroxide and 2% potassium ferrocyanide. After rinsing with deionized water (18.25 MΩ), samples were dehydrated through a graded acetone series (30%, 50%, 70%, 80%, and 95% for 10 min each, followed by two changes in 100% acetone for 20 min each). The dehydrated tissues were infiltrated with SPI resin using acetone–resin mixtures (3:1 and 1:1, *v*/*v*) at 37 °C and then embedded in pure resin overnight. Polymerization was carried out at 60–69 °C for 48 h.

Ultrathin sections (70–90 nm) were prepared using a Leica UC7 ultramicrotome (Leica Microsystems GmbH, Wetzlar, Germany), stained with uranyl acetate and lead citrate, and observed under a Hitachi HT7800 transmission electron microscope (Hitachi High-Tech Corporation, Minato-ku, Tokyo, Japan). Ultrastructural differences between control and ds*MaCht-2*-treated samples were compared with particular attention to cuticle layering, lamellar organization, and overall structural integrity.

## 3. Results

### 3.1. Molecular Characterization of MaCht-2

**Figure 1:**
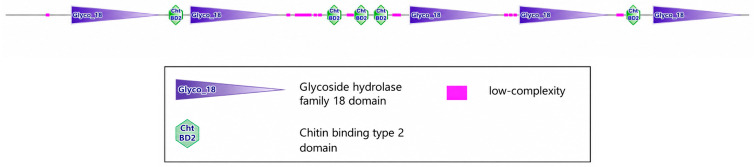
Domain organization of *MaCht-2*.

To clarify the molecular characteristics of *MaCht-2*, its full-length cDNA sequence was analyzed and the encoded protein structure was predicted. The results showed that *MaCht-2* contained five Glyco_18 catalytic domains and five chitin-binding domains (ChtBD2), indicating that it possesses the conserved domain architecture typical of insect chitinases (Figure 1). This multi-domain organization suggests that *MaCht-2* may play an important role in chitin recognition and cuticle remodeling.

**Figure 2:**
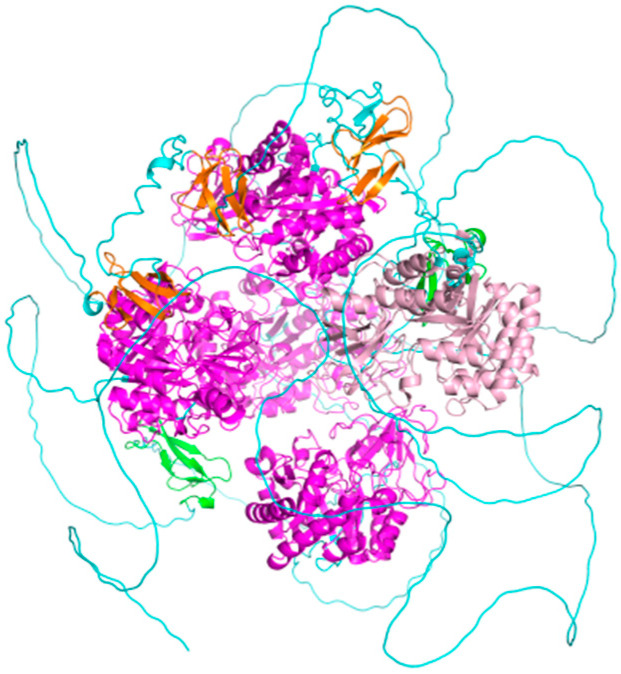
Predicted three-dimensional structure of *MaCht-2*. GH18 chitolectin chitotriosidase is colored pink, while the GH18 chitinase-like superfamily is colored light pink. Due to structural similarity to the GH18 chitolectin chitotriosidase domain, a similar color was chosen for annotation. ChtBD2 is colored yellow, and CBM 14 is colored green.

The deduced protein was predicted to comprise 8274 amino acids, with a theoretical molecular weight of 718,610.43 Da and a predicted isoelectric point of 4.60. Three-dimensional structure prediction further showed that *MaCht-2* contained conserved GH18 chitinase-like regions and chitin-binding modules, including *ChtBD2* and *CBM14* (Carbohydrate-Binding Module family 14), consistent with the structural characteristics of insect *Cht2* proteins (Figure 2). Overall, these molecular features support the classification of *MaCht-2* as a conserved chitinase family member and provide a structural basis for its involvement in molting and cuticle formation in *M. alternatus*.

### 3.2. Developmental Expression Profile of the MaCht-2

**Figure 3:**
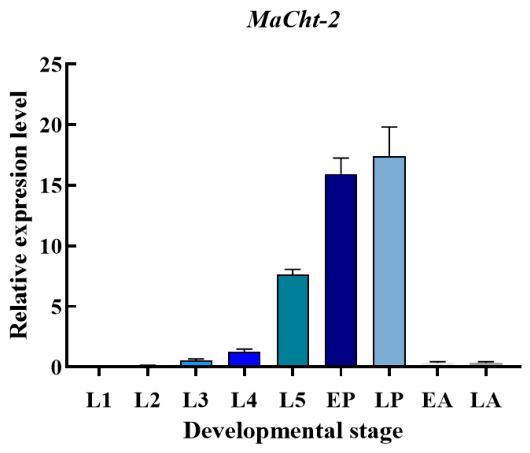
*MaCht-2* expression profiles across developmental stages of *M. alternatus*.

The temporal expression profile of *MaCht-2* across developmental stages of *M. alternatus* was examined using qPCR. The results indicated that *MaCht-2* transcript levels remained relatively low during the early larval stages (L1–L4). Expression increased markedly at the fifth instar larval stage (L5) and peaked during both the early and late pupal stages (EP and LP), before declining sharply in the adult stages (EA and LA) (Figure 3).

Quantitative assessment showed that transcript abundance at the L5 and pupal stages was significantly higher than that observed in earlier larval stages (*p* < 0.05). The concentration of high transcript abundance within the L5-to-LP interval indicates that *MaCht-2* is developmentally regulated and mainly active during periods of intense cuticle remodeling and metamorphic reorganization. This continuous upregulation across the late larval and pupal stages further suggests that *MaCht-2* may participate in a broad developmental window associated with molting and cuticle reconstruction.

### 3.3. RNAi Efficiency and Temporal Dynamics of Gene Silencing

To assess the silencing efficacy of dsRNA targeting *MaCht-2*, fifth-instar larvae were administered three doses of dsRNA (5 µg, 10 µg, and 15 µg), and transcript levels were measured at 12, 24, and 48 h post-injection using qPCR. Individuals injected with dsGFP were used as controls.

**Figure 4:**
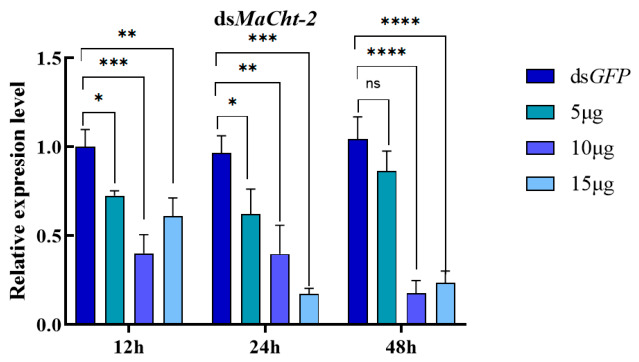
Changes in chitinase gene expression following ds*MaCht-2* injection. Note: ns represents *p* > 0.05; * Indicating *p* < 0.05; ** Indicating *p* < 0.01; *** Indicates *p ≤* 0.001; **** Indicates *p* < 0.0001.

At 12 h post-injection, all ds*MaCht-2* treatments resulted in a significant reduction in *MaCht-2* transcript levels compared with the control group (*p* < 0.05), indicating rapid activation of RNAi-mediated suppression (Figure 4). Among the treatments, the 10 µg dose exhibited the greatest level of suppression, followed by the 15 µg and 5 µg treatments.

At 24 h, *MaCht-2* expression remained markedly reduced in all treatment groups. By 48 h, transcript levels in the 5 μg group had recovered to a level not significantly different from that of the control (*p* > 0.05), whereas the 10 μg and 15 μg groups still maintained substantial suppression. Thus, although all tested doses triggered a rapid RNAi response, the persistence of gene silencing differed among treatments, with medium and high doses producing a longer-lasting effect than the low dose.

Taken together, these results indicate that *MaCht-2* is sensitive to RNAi-mediated knockdown and that the duration of transcript suppression is strongly influenced by dsRNA dosage.

### 3.4. RNAi-Induced Phenotypes and Dose-Dependent Effects

**Figure 5:**
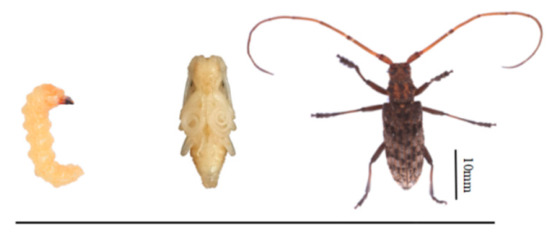
Normal phenotype of the control group of *M. alternatus* injected with dsGFP.

**Figure 6:**
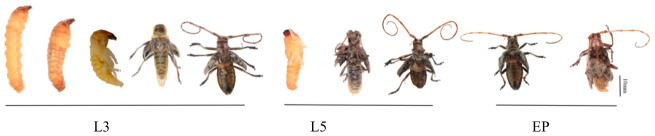
Phenotype outcomes of *M. alternatus* following varying doses of ds*MaCht-2* injection at different developmental stages (L3, L5, and EP indicate the developmental stages of *M. alternatus* at which dsRNA was injected).

Injection of dsGFP caused no visible developmental abnormalities in *M. alternatus* (Figure 5). In contrast, ds*MaCht-2* treatment at different developmental stages produced pronounced defects related to molting, metamorphosis, and adult formation. Both the severity and the timing of these phenotypes were dependent on the developmental stage at injection and on dsRNA dosage (Figure 6; Table 2).

#### 3.4.1. Phenotypic Effects in Third-Instar Larvae

Following ds*MaCht-2* administration at the third-instar stage, most individuals were unable to complete normal molting and perished during ecdysis. The treated larvae displayed darkened cuticle pigmentation and evident separation of the old larval cuticle, revealing incomplete shedding despite the initiation of a new cuticle (Figure 6).

Larval mortality increased with dsRNA dosage, reaching 54.55%, 63.64%, and 84.62% in the 5 µg, 10 µg, and 15 µg treatment groups, respectively (Table 2). Survivors often develop into malformed pupae that retain larval traits or emerge as severely deformed adults. Affected individuals displayed complete impairment of locomotor activity across all dosage groups. These results indicate that *MaCht-2* knockdown in early larvae causes strong dose-dependent lethality and severe disruption of normal molting.

#### 3.4.2. Phenotypic Effects in Fifth-Instar Larvae

In contrast, low-dose ds*MaCht-2* treatment (5 µg) did not impede the successful pupation of fifth-instar larvae; however, all emerged adults exhibited severe morphological defects. The observed phenotypes included the persistence of the pupal cuticle, incomplete wing expansion, aberrant antenna curvature, and reduced attachment ability to surfaces (Figure 6).

As the dsRNA dosage increased, developmental perturbations manifested at earlier stages. In the 15 µg treatment group, approximately 50% of individuals experienced molting failure during the larval–pupal transition, resulting in incomplete shedding of the larval cuticle and subsequent death before or during adult emergence. Individuals who successfully emerged still exhibited severe morphological deformities. Overall, locomotor function was compromised across all treatments, and mortality occurred at earlier time points with higher dsRNA concentrations. Thus, increasing the extent of *MaCht-2* suppression shifted the phenotype from adult deformity to earlier molting failure.

#### 3.4.3. Phenotypic Effects of Pupal Injection

Injection of ds*MaCht-2* at the pupal stage mainly influenced wing morphogenesis. The treated individuals typically displayed incomplete pupal cuticle shedding, accompanied by malformed or wrinkled wings upon emergence (Figure 6).

The proportion of adults displaying impaired locomotion increased with the dsRNA dosage, reaching 100% in the 15 µg treatment group. The incidence of adult deformities progressively increased, reaching 20%, 60%, and 70% in the 5 µg, 10 µg, and 15 µg groups, respectively (Table 2). These findings show that *MaCht-2* is also required during pupal development and that its silencing disrupts late metamorphic remodeling in a dose-dependent manner. The frequent occurrence of cuticle retention, malformed appendages, and reduced mobility suggested that *MaCht-2* silencing may severely impair cuticle organization, which was further examined by TEM.

### 3.5. Ultrastructural Cuticle Alterations After MaCht-2 Silencing

**Figure 7:**
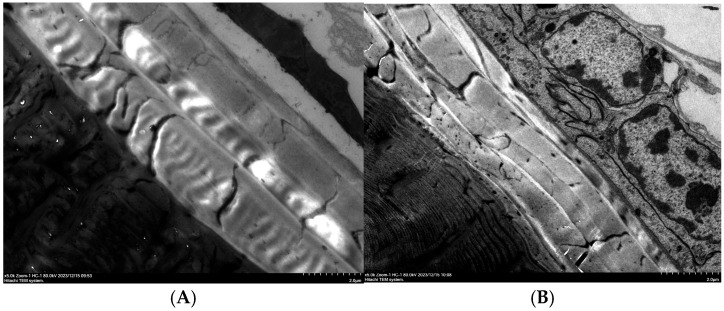
Ultrastructure of the sheath wing epidermis in the control group (**A**) and after *MaCht-2* silencing (**B**).

**Figure 8:**
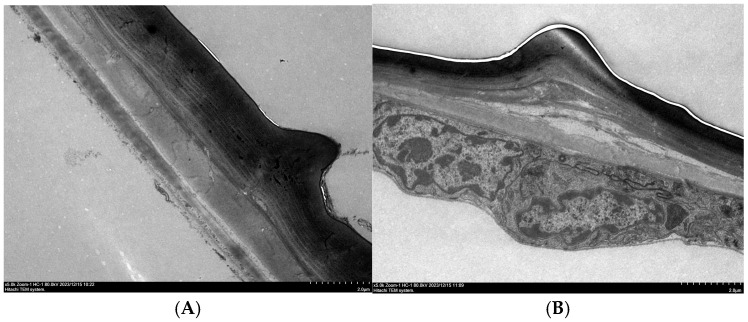
Ultrastructure of the abdominal epidermis before (**A**) and after *MaCht-2* silencing (**B**).

Transmission electron microscopy was performed to evaluate cuticle ultrastructure in adults that emerged after ds*MaCht-2* treatment. Elytral and abdominal epidermal tissues from treated individuals were compared with those from dsGFP-injected controls. In the control group, both tissues displayed a smooth and regularly arranged lamellar structure with clearly defined cuticular layers (Figure 7A and Figure 8A). By contrast, the cuticle of ds*MaCht-2*-treated insects was evidently thinner, more wrinkled, and more disorganized in both the elytral and abdominal epidermis (Figure 7B and Figure 8B).

The lamellar arrangement in treated individuals was markedly disrupted, indicating impaired cuticle formation and reduced structural integrity after *MaCht-2* silencing. These ultrastructural abnormalities were consistent with the severe molting defects and adult deformities observed in the RNAi-treated insects. Together, these results provide direct morphological evidence that *MaCht-2* is essential for normal cuticle organization in *M. alternatus*.

## 4. Discussion

### 4.1. Role of MaCht-2 in Molting and Metamorphosis

Chitinases are essential for insect molting and metamorphosis because they participate in the degradation of old cuticle and the remodeling of newly formed cuticular structures [8,11]. In this study, *MaCht-2* showed a distinct developmental expression pattern, with low transcript abundance during early larval stages and strong upregulation from the fifth-instar larval stage to the pupal stage. This sustained elevation across the L5-to-LP interval corresponds to a period of active cuticle turnover and metamorphic reorganization in *M. alternatus*, indicating that *MaCht-2* is closely associated with developmental remodeling. Similar stage-biased expression of chitinase genes has been reported in other insects, especially during periods of intensive cuticle reconstruction and eclosion-related development [11,20].

Functional evidence from RNAi further demonstrated the importance of *MaCht-2* in these processes. Silencing of *MaCht-2* caused severe defects across multiple developmental stages, including incomplete larval ecdysis, failed larval–pupal transition, retention of pupal cuticle, malformed wings, and impaired adult locomotion. These phenotypes indicate that *MaCht-2* is required not only for successful removal of the old cuticle, but also for proper formation and maturation of the new cuticle. Because comparable defects were induced by dsRNA administration at the larval and pupal stages, *MaCht-2* is likely involved in a broad developmental window rather than in a single stage-specific event. This interpretation is consistent with previous studies showing that disruption of chitin metabolism-related genes frequently leads to incomplete ecdysis, cuticle defects, and developmental arrest [8,20].

Moreover, the ultrastructural abnormalities observed after *MaCht-2* knockdown, including thinning, wrinkling, and disorganization of the cuticular lamellae, provide morphological support for its role in maintaining cuticle integrity. Proper lamellar organization is a key feature of normal insect cuticle differentiation, and disruption of this architecture is commonly associated with defective cuticle maturation and impaired mechanical function [12]. Taken together, these results indicate that *MaCht-2* is a key regulator of molting and metamorphosis in *M. alternatus* and is likely required for coordinated cuticle remodeling during successive developmental transitions.

### 4.2. RNAi Efficiency and Dose-Dependent Gene Silencing

RNA interference has become an important tool in insect functional genomics and has considerable potential for the development of environmentally friendly pest control strategies [14,15]. In the present study, *MaCht-2* transcript levels were significantly reduced as early as 12 h after dsRNA injection, indicating that RNAi-mediated suppression was rapidly activated in *M. alternatus*. This rapid response suggests that injected dsRNA can be efficiently recognized and processed in this species, at least under laboratory conditions. Similar rapid or early onset of gene silencing after dsRNA administration has been reported not only in diverse insects in general [14,15], but also in *M. alternatus*, where dsRNA injection against CHS1 or TDO led to marked transcript reduction within the first 48 h [22,23].

A clear dose-dependent pattern was observed in the persistence of *MaCht-2* knockdown. Although all three tested doses caused significant suppression at 12 h, transcript levels in the 5 μg treatment recovered to near-control levels by 48 h, whereas the 10 μg and 15 μg treatments maintained substantial suppression over the same period. This pattern indicates that the biological outcome of RNAi in *M. alternatus* depends not only on whether gene silencing is initiated, but also on how long transcript depletion can be sustained. Previous studies have shown that RNAi efficiency in insects is influenced by multiple factors, including dsRNA uptake, intracellular transport, nuclease-mediated degradation, and the inherent susceptibility of the target gene and tissue [14,15] and, under applied settings, by dsRNA formulation and environmental stability as well [14,15,24]. Therefore, the prolonged suppression observed at the medium and high doses in this study likely reflects a stronger and more persistent RNAi effect rather than a simple transient knockdown.

Importantly, the difference in silencing persistence was consistent with the subsequent phenotypic outcomes. Lower-dose treatment tended to permit progression through at least part of development, although with severe adult abnormalities, whereas higher-dose treatment more frequently caused earlier developmental failure, including lethal molting defects. This shift suggests that the extent of *MaCht-2* suppression influences not only phenotype severity but also the developmental stage at which defects become manifested. A similar distinction between early transcript suppression and later biological consequences has also been noted in other cerambycid RNAi studies, where knockdown persisted over several days before stable behavioral or developmental phenotypes became fully apparent [23,25]. In other words, transient knockdown may be sufficient to impair later cuticle maturation, whereas stronger and longer-lasting suppression is more likely to disrupt earlier molting transitions. Such stage-dependent consequences of RNAi intensity are consistent with the broader view that effective target-gene selection for pest control must consider not only transcript reduction itself, but also the durability and biological impact of that reduction [13]. It should be noted that the current study did not include a non-injected or buffer-only blank control group, following the common practice of using dsGFP-injected insects as the negative control in *M. alternatus* RNAi studies. Although all dsGFP-injected individuals developed normally, the inclusion of an additional blank control in future experiments would help to further isolate the potential effects of the injection procedure. In addition, because gene expression was monitored only up to 48 h post-injection, whereas phenotypic defects were observed through later developmental stages, it remains unclear whether the adult-stage phenotypes resulted from sustained transcript suppression or from a cascade triggered by early transient silencing. Future studies with larger sample sizes and extended expression monitoring at later time points, such as during the prepupal or pre-eclosion period, would be valuable to clarify this relationship.

Taken together, these findings indicate that *MaCht-2* is highly responsive to RNAi-mediated knockdown in *M. alternatus*, and that dsRNA dosage strongly affects the duration of transcript suppression and the severity of downstream developmental defects. Recent discussions of RNAi target discovery have emphasized that effective targets should combine strong transcript responsiveness with durable and biologically meaningful phenotypes, a conclusion also supported by recent screening-based studies of cuticle-associated genes [13,26].

### 4.3. Functional Differences Among Chitin-Related Genes

Functional divergence within the insect chitin metabolism network has been increasingly recognized, and accumulating evidence indicates that not all chitin-related genes contribute equally to molting control, cuticle maintenance, or developmental survival [27,28,29]. Genome-wide and family-level analyses in multiple insect species have shown that chitinase genes differ substantially in expression timing, tissue distribution, and biological function, with only a subset exhibiting strong developmental indispensability when silenced [30]. For example, in *Sogatella furcifera*, comprehensive analysis of the chitinase family revealed that although several genes were expressed in the integument and peaked before molting, only selected members such as SfCht5, SfCht10, and SfIDGF2 produced severe molting defects and lethality after RNAi, highlighting marked functional specialization within the same pathway [30].

The results of the present study are consistent with this broader pattern of gene-specific functional differentiation. Compared with previously characterized chitin-related genes in *M. alternatus*, *MaCht-2* caused stronger and more consistent developmental disruption, including high larval mortality, severe molting failure, retention of old cuticle, malformed adults, and widespread locomotor impairment. This suggests that *MaCht-2* may occupy a more critical or less functionally redundant position in cuticle remodeling than some other previously tested targets. Although multiple genes participate in chitin turnover, their biological importance is clearly not equivalent, and the severity of the phenotype after RNAi may reflect how centrally a given gene contributes to developmental transitions.

Evidence from other insect systems also supports the view that *Cht2*-like genes can have major developmental importance. In *Musca domestica*, knockdown of *MdCht2* caused high mortality and abnormal eclosion, indicating that Group VII/*Cht2* genes can be essential for successful metamorphosis [20]. More broadly, studies in other insects have shown that disruption of chitin metabolism regulators can differentially affect the expression of downstream chitinase genes and lead to distinct phenotypic outcomes, ranging from incomplete ecdysis to wing deformities and developmental arrest [31,32]. These findings emphasize that chitin metabolism should be viewed as a coordinated but functionally heterogeneous network rather than as a set of interchangeable targets.

From an applied perspective, these differences are particularly important for RNAi-based pest management. Recent work has argued that the most useful RNAi targets are not simply genes that can be knocked down at the transcript level, but genes whose suppression produces strong, durable, and biologically consequential phenotypes [13]. In this context, *MaCht-2* appears to be a comparatively promising target in *M. alternatus* because its silencing consistently caused both lethal and sublethal defects across multiple developmental stages. Such a phenotype profile may be especially valuable in vector management, where both mortality and severe impairment of adult quality may contribute to control efficacy.

When compared with our previously reported data on *MaCht-3* and *MaIDGF-4*, which were obtained under the same laboratory conditions and with dsRNA prepared by identical methods, the phenotypic differences are evident. For third-instar larvae, injection of 5 µg ds*MaCht-3* produced no larval mortality and limited malformation, whereas the same dose of ds*MaCht-2* caused 54.55% larval mortality. Similarly, ds*MaIDGF-4* at 5 µg and 10 µg resulted in no larval death in third-instar larvae or produced mortality only inconsistently across doses, in contrast to the clear dose-dependent lethality observed for *MaCht-2*. Severe malformation rates and the incidence of complete locomotor impairment were also consistently higher after *MaCht-2* silencing than after knockdown of the two previously characterized genes. Although a formal statistical meta-analysis across studies is precluded by differences in experimental design and sample sizes, the overall pattern supports the conclusion that *MaCht-2* is a comparatively high-impact RNAi target within the chitin metabolic network of *M. alternatus*.

### 4.4. Ultrastructural Evidence of Cuticle Disruption

The TEM observations in this study provide direct structural evidence that *MaCht-2* is required for normal cuticle organization in *M. alternatus*. In control insects, the elytral and abdominal cuticles displayed a regular lamellar architecture with clearly defined layers, which is characteristic of properly differentiated insect cuticle [12]. By contrast, *MaCht-2*-silenced individuals exhibited obvious cuticular abnormalities, including thinning, wrinkling, and disorganization of the lamellar layers. These results indicate that *MaCht-2* is required not only for developmental progression at the whole-organism level, but also for maintaining the structural integrity of the cuticle at the ultrastructural level.

The biological significance of these ultrastructural changes is likely substantial. The layered organization of the insect cuticle is closely associated with its mechanical stability and functional performance, and disruption of this organization can compromise the ability of the cuticle to support normal molting, appendage expansion, and locomotion [12,33]. In this study, the disrupted lamellar arrangement observed after *MaCht-2* knockdown was consistent with the high frequency of incomplete ecdysis, retained cuticle, wing deformities, and reduced mobility. Thus, the TEM results help explain how transcript-level suppression of *MaCht-2* was translated into severe organismal phenotypes. Compared with previously characterized chitin-related genes in *M. alternatus* for which comparable injection-based RNAi data are available, *MaCht-2* showed higher larval mortality and a greater proportion of severe morphological defects, supporting its potential value for target prioritization in RNAi-based control. The observed locomotion defects were based on qualitative assessment, and formal behavioral quantification is needed to confirm the extent of functional impairment. Further studies should also evaluate alternative dsRNA delivery strategies and assess the practical applicability of *MaCht-2* targeting under more realistic conditions.

Comparable ultrastructural defects have been reported in other insects after perturbation of chitin metabolism-related genes. In *Tribolium castaneum*, disruption of chitinase and imaginal disc growth factor genes altered chitin extracellular matrix organization and deformed the lamellate procuticle, while knockdown of TcCHT7 impaired the organization of chitin in newly forming cuticle rather than simply affecting degradation of the old cuticle [19,34]. Likewise, studies targeting chitin synthase-related pathways have shown that defective cuticle deposition is often accompanied by thin, poorly organized, and weakly compacted cuticular layers [35]. Together, these reports support the interpretation that the ultrastructural defects observed here are a direct manifestation of disturbed cuticle remodeling after *MaCht-2* silencing.

An additional implication of these findings is that *MaCht-2* may contribute to cuticle organization more broadly than would be expected from a role limited to old-cuticle degradation alone. Because structural disruption was observed in both the elytral and abdominal epidermis, *MaCht-2* likely participates in the coordinated formation and maturation of new cuticle in multiple body regions. This broader structural role is consistent with the severe developmental consequences of *MaCht-2* knockdown and further strengthens its importance as a candidate molecular target in *M. alternatus*.

## 5. Conclusions

*MaCht-2* is essential for normal molting and cuticle formation in *Monochamus alternatus*. Its expression was markedly upregulated from the fifth-instar larval stage to the pupal stage, indicating a close association with periods of active cuticle remodeling. RNAi-mediated silencing of *MaCht-2* caused severe developmental defects, including incomplete ecdysis, failed larval–pupal transition, adult malformation, and impaired locomotion. TEM analysis further showed that *MaCht-2* knockdown disrupted cuticle ultrastructure, resulting in thinning, wrinkling, and disorganized lamellar arrangement. Together, these results demonstrate that *MaCht-2* is required for coordinated cuticle remodeling during development and represents a promising RNAi target in *M. alternatus*.

Compared with previously characterized chitin-related genes in *M. alternatus*, *MaCht-2* showed stronger and more consistent RNAi-induced effects, supporting its potential value for target prioritization in RNAi-based control. Further studies should evaluate alternative dsRNA delivery strategies and assess the practical applicability of *MaCht-2* targeting under more realistic conditions.

## Figures and Tables

**Table 1:** Primer information.

Primers	Primer Sequences 5′–3′
*Actin-F*	TGGGTATGGAATCTTGCGGT
*Actin-R*	GGCCCTGATTTCCTTTTGCA
*MaCht-2-F*	CCCCTGCTTCCAGGTTTAGG
*MaCht-2-R*	GATCCGCTACCGGTTCTCTG
*dsMaCht-2-F*	TCCCAGCGACAAATATCCTC
*dsMaCht-2-R*	AACGTAAAAGCGGGAAGGTT
*dsMaCht-2-T7F*	GGATCCTAATACGACTCACTATAGGGTCCCAGCGACAAATATCCTC
*dsMaCht-2-T7R*	GGATCCTAATACGACTCACTATAGGGAACGTAAAAGCGGGAAGGTT
*dsGFP-F*	ACGTAAACGGCCACAAGTTC
*dsGFP-R*	TGTTCTGCTGGTAGTGGTCG
*dsGFP-T7F*	GGATCCTAATACGACTCACTATAGGGACGTAAACGGCCACAAGTTC
*dsGFP-T7R*	GGATCCTAATACGACTCACTATAGGGTGTTCTGCTGGTAGTGGTCG

**Table 2:** Rates of affected individuals following dsRNA injections in *M. alternatus*.

Target Gene	Interference Phase	Injection Dose (μg)	Larval Mortality Rate%	Pupal Mortality %	Severe Malformation in Adult Insects %	Slight Malformation in Adult Insects %	Impact on Mobility %	Infection Fatality %
*MaCht-2*	3rd instar larvae	5	54.55	0	45.45	0	100	0
		10	63.64	0	36.36	0	100	0
		15	84.62	0	15.38	0	100	0
	4th-5th instar larvae	5	0	0	100	0	100	42.86
		10	25	0	75	0	100	20
		15	25	37.5	37.5	0	100	0
	pupa	5	0	0	20	20	20	0
		10	0	0	60	20	60	0
		15	0	30	70	0	100	0
*MaCht-3*	3rd instar larvae	5	27.27	0	27.27	27.27	54.55	0
		10	0	0	30	50	30	0
		15	0	0	30	50	30	0
	4th-5th instar larvae	5	0	0	16.67	50	16.67	0
		10	0	0	40	20	40	0
		15	0	0	80	20	80	0
	pupa	5	0	0	33.33	50	33.33	0
		10	0	0	100	0	100	0
		15	0	0	80	20	80	0
*MaIDGF-4*	3rd instar larvae	5	0	0	10	70	10	0
		10	50	0	0	25	50	33.33
		15	0	0	70	30	70	0
	4th-5th instar larvae	5	0	0	50	0	50	14.29
		10	25	0	50	0	75	20
		15	0	0	50	50	50	20
	pupa	5	0	20	30	40	50	23.08
		10	0	0	85.71	14.29	85.71	0
		15	0	28.57	57.14	14.29	85.71	0

## Data Availability

The original contributions presented in this study are included in the article. Further inquiries can be directed to the corresponding author.
